# Army Medical Intelligence—Official

**Published:** 1864-11

**Authors:** 


### ARMY MEDICAL INTELLIGENCE—OFFICIAL.

### LIST OF MEDICAL PURVEYORS, C. S. ARMY.

| | | | |
|---|---|---|---|
| Surgeon | E. W. Johns, C. S. A. | | Richmond, Va. |
| " | J. S. Johnson, P. A. C. S. | | Charlotte, N. C. |
| " | J. J. Chisolm, | " | Columbia, S. C. |
| " | W. H. Priolean, | " | Macon, Ga. |
| " | G. S. Blackie, | " | Augusta, Ga. |
| " | W. H. Anderson, | " | Montgomery, Ala. |
| " | R. Miller, | " | Mobile, Ala. |
| " | R Potts, | " | Montgomery, Ala. |
| " | R. K. Taylor, | " | Lynchburg, Va. |
| " | J. E. A. Davidson, | " | Quincy, Fla. |
| " | H. P. Howard, | " | San Antonia, Texas. |
| " | J. P. Bond, | " | Camden, Ark. |
| " | H. Stockdell, | " | Wilmington, N. C. |
| " | W. H. Geddings, | " | Army of N. Virginia. |
| " | W. C. Boon, | " | Bonham, Texas. |
| " | R. M. Sutfield, | " | Columbus, Ga. |
| " | John Clopton, | " | Danville, Va. |
| " | Howard Smith, | " | Houston, Texas. |
| " | E. H. C. Bailey, | " | Demopolis, Ala. |
| " | Thos. Liming, | " | Charleston, S. C. |
| " | J. W. Hines, | " | Richmond, Va. |
| " | Bushrod Taylor, | " | Army of Valley Dist. |
| Ass't Surg. | R. Q. Stoney, | " | Savannah, Ga. |
| " | W. Morrow, | " | Army of Tennessee. |
| " | W. B. Robertson, | " | Wytheville, Va. |
| " | J. C. Stickney, | " | Columbus, Ga. |
| " | Z B. Herndon, | " | Lake City, Fla. |
| " | J. F. Young, | " | Columbus, Ga. |
| " | W. H Baldwin, | " | Houston, Texas. |
| " | P. J. Johnson, | " | Alexandria, La. |
| " | H C. Rogers, | " | Shreveport, La. |
| " | E. Cross, | " | Doaksville, C. N. |

